# Land management strategies can increase oil palm plantation use by some terrestrial mammals in Colombia

**DOI:** 10.1038/s41598-019-44288-y

**Published:** 2019-05-24

**Authors:** Lain E. Pardo, Mason J. Campbell, Michael V. Cove, Will Edwards, Gopalasamy Reuben Clements, William F. Laurance

**Affiliations:** 1 School of Natural Resource Management, Nelson Mandela University, George Campus, Madiba Drive 6530, George, South Africa; 2 Centre for Tropical Environmental and Sustainability Science (TESS), College of Science and Engineering, James Cook University, Cairns, Queensland 4878 Australia; 3 Department of Applied Ecology, North Carolina State University, Raleigh, North Carolina 27695 USA; 4 Department of Biological Sciences, Sunway University, 47500 Bandar Sunway, Selangor, Malaysia; 5 Rimba, Jalan Kiara 5, 50480 Kuala, Lumpur Malaysia

**Keywords:** Conservation biology, Agroecology, Ecological modelling

## Abstract

While the conservation role of remaining natural habitats in anthropogenic landscapes is clear, the degree to which agricultural matrices impose limitations to animal use is not well understood, but vital to assess species’ resilience to land use change. Using an occupancy framework, we evaluated how oil palm plantations affect the occurrence and habitat use of terrestrial mammals in the Colombian Llanos. Further, we evaluated the effect of undergrowth vegetation and proximity to forest on habitat use within plantations. Most species exhibited restricted distributions across the study area, especially in oil palm plantations. Habitat type strongly influenced habitat use of four of the 12 more widely distributed species with oil palm negatively affecting species such as capybara and naked-tailed armadillo. The remaining species showed no apparent effect of habitat type, but oil palm and forest use probabilities varied among species. Overall, generalist mesocarnivores, white-tailed deer, and giant anteater were more likely to use oil palm while the remaining species, including ocelot and lesser anteater, showed preferences for forest. Distance to nearest forest had mixed effects on species habitat use, while understory vegetation facilitated the presence of species using oil palm. Our findings suggest that allowing undergrowth vegetation inside plantations and maintaining nearby riparian corridors would increase the likelihood of terrestrial mammals’ occurrence within oil palm landscapes.

## Introduction

Agricultural expansion is one of the major drivers of global habitat fragmentation and habitat loss^1,2^. This process has had clear negative effects on biodiversity worldwide^2–4^. However, the intensity of the effects of land-use change on native fauna depends on the type of agriculture replacing natural ecosystems, as well as other landscape or local factors such as the type of crop, the total area of remaining vegetation and its spatial configuration and the potential availability of new resources^5–7^. Some agroecosystems, for example, can facilitate the occurrence of generalist species^8–10^, while others may act as barriers or ecological traps with elevated risk of mortality for rare and specialized species^11,12^. Therefore, understanding how agroecosystems limit animal movement or occurrence is fundamental for determining species’ survival under increasing agricultural expansion.

Oil palm has become one of the most important agricultural products in the world. However, the increased cultivation of this crop has had negative effects on the biodiversity of tropical regions such as Southeast Asia^13,14^. Oil palm cultivation is increasing in Latin America (Neotropics)^15,16^, where Colombia is the largest producer with ~500,000 ha currently under cultivation^17^. Contrary to Southeast Asia, most recent Latin-American and Colombian oil palm expansion has taken place in previously transformed lands^16,18^; which has minimized the impact on biodiversity. Nevertheless, there is concern that oil palm plantations will expand into natural areas, especially in savannas of the eastern Llanos region, or Orinoco Basin^18–20^.

Worldwide, geographic ranges of mammal species have reduced by ~30%, especially in locations of intensive human activities (e.g. agriculture and settlements)^21^. Colombia is globally ranked among the five most mammal diverse countries, with 518 species recorded to date^22^. However, little is known about the natural history and distribution of most of Colombia’s terrestrial mammals^23,24^, especially in human-dominated landscapes. The lack of mammal studies (especially camera trapping) is particularly evident in the Llanos and Amazon regions^25^ despite the Llanos region containing ~68% of the Colombian mammal diversity (including Chiroptera)^26^. This region has also become the most attractive area for implementation of agribusiness development (such as oil palm) in Colombia^18,27^. The western Llanos, for example, is a large area composed of different ecosystems, including savanna, wetlands and “morichales” (native palm agglomerations), mixed systems of native and introduced grassland and riparian forest^28^. This mixed system or mosaics has suffered a long history of disturbance by several agricultural processes, particularly grazing, that have cleared savannas and reduced the width of riparian forests^19,29^. After pastures, oil palm is currently the most important crop in this region of western Llanos and it is predicted to continue expanding^27^.

Evaluating the probability that a species will occupy areas of different land-uses provides an estimation of the degree of resistance (or permeability) imposed by the surrounding agricultural matrix. In other words, how difficult is it for an animal to travel through the matrix? Previous studies in other taxa have shown that proximity to riparian forest increase spillover rates from forest to oil palm by increasing the probability of animal species (e.g. beetles, ants, birds) to occur in oil palm matrixes (e.g.^30–33^). Although, investigations into the potential impacts of oil palm production on Colombian fauna have recently increased^32,34–37^, there is still a paucity of information evaluating mammal species responses to oil palm plantations. This information is important, since the capacity of protected areas to function as a mechanism for biodiversity conservation in the long-term is limited, given the increasing rate of degradation occurring in these areas^38,39^, and the surrounding landscapes^40,41^.

Due to the rapid expansion of oil palm in Colombia, there is an urgent need to assess the ability of mammals to tolerate and traverse the matrix of oil palm cover and to identify management practices that can reduce its biodiversity impacts. Here, we compared the habitat use of terrestrial mammals between riparian forest and oil palm plantations in the eastern plains of Colombia. In this way, we evaluated whether the oil palm matrix acts as a movement barrier (or habitat filter) for these species and, if so, the degree to which this occurs. Further, we determined whether local habitat variables and management regimes: distance to forest and the presence of undergrowth vegetation, increased the likelihood of species occurrence within oil palm habitats.

## Results

### General patterns of mammalian distribution in the study area

**Figure 1:**
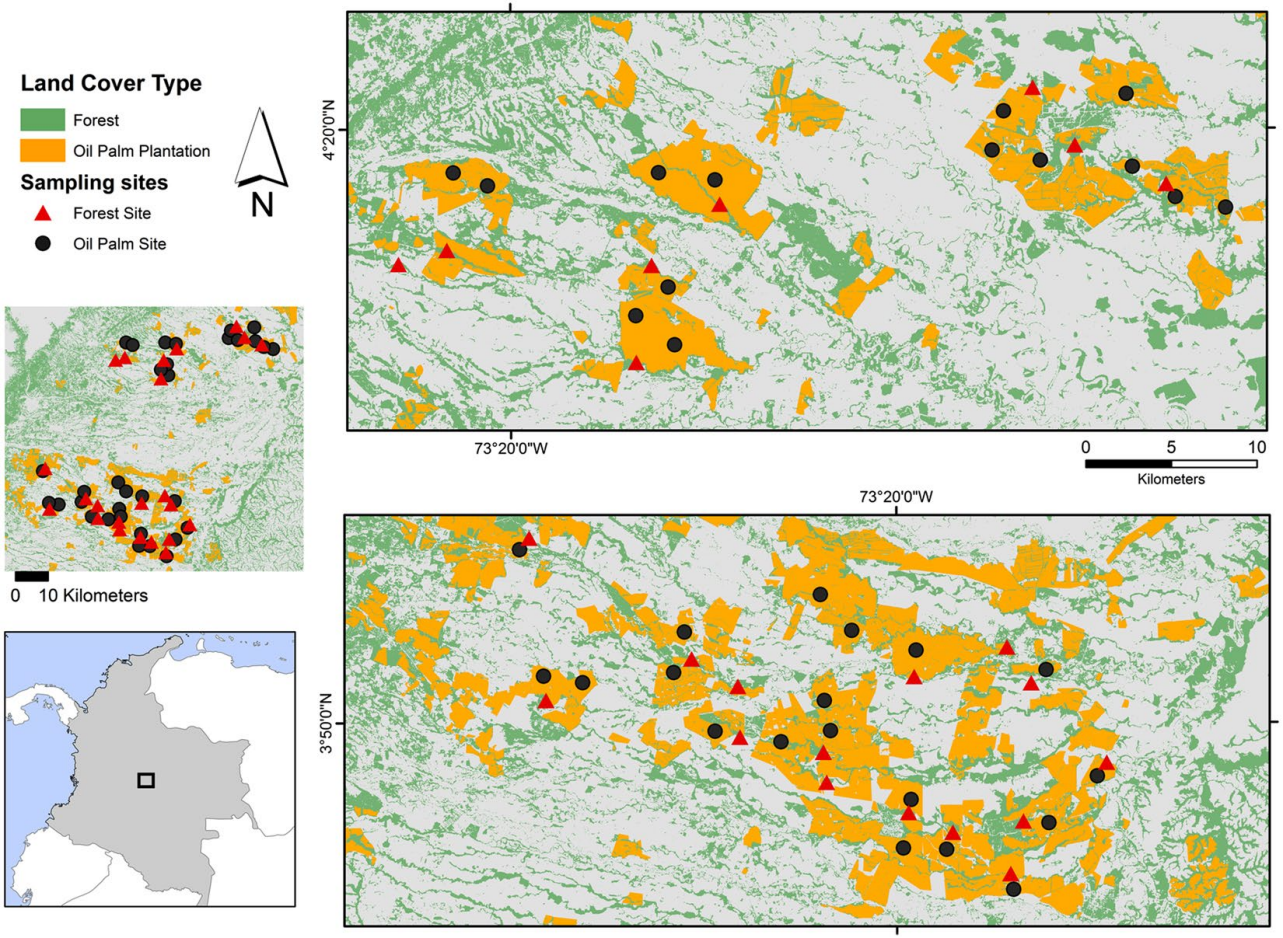
Study area and location of sample sites (n = 56) across the Llanos region in Colombia (Grey area indicates other land-uses, mainly pasture).

Twenty-three terrestrial species of medium and large mammals and two species of arboreal primates were detected across the study area (Fig. 1, Table 1, see^37^ for details). Most of the species in the study area were rare in the landscape and only five species were detected at more than 50% of all surveyed sites (naïve occupancy without accounting for detectability). These were: giant anteater (*Myrmecophaga tridactyla*), lesser anteater (*Tamandua tetradactyla*), crab-eating fox–here after fox (*Cerdocyon thous*), ocelot (*Leopardus pardalis*), and common opossum (*Didelphis marsupialis*).

**Table 1:** Naïve occupancy, model-averaged estimates of overall habitat use $\hat{{\boldsymbol{\Psi }}})$ and habitat preference of 23 terrestrial mammals detected in oil palm-dominated landscapes in the Llanos region, Colombia. Positive Beta values suggest preference for oil palm and negative values suggest preference for forest.

Common name	Scientific name	Occupied sites		Naive Occupancy			Overall estimated occupancy			Habitat preference
		Forest (n = 23)	Oil Palm (n = 33)	Study area	Forest	Oil Palm	Est. Ψ (Sd) study area	Est. Ψ (Sd) forest	Est. Ψ (Sd) oil palm	Beta oil palm (SE)
Giant anteater	*Myrmecophaga tridactyla*	19	30	0.88	0.83	0.91	0.90 (0.04)	0.86 (0.07)	0.94 (0.06)	2.12 (2.60)
Lesser anteater	*Tamandua tetradactyla*	20	16	0.64	0.87	0.48	0.72 (0.16)	0.90 (0.13)	0.59 (0.17)	−1.64 (0.97)
Nine-banded armadillo	*Dasypus novemcinctus*	16	3	0.34	0.70	0.09	0.57 (0.10)	0.69 (0.09)	0.49 (0.25)	−2.32 (1.15)
Naked-tailed armadillo	*Cabassous unicinctus*	9	0	0.16	0.39	0.00	0.19 (0.23)	0.47 (0.13)	0	—
Crab-eating fox	Cerdocyon thous*	6	26	0.57	0.26	0.79	0.60 (0.28)	0.27 (1.71)	0.82 (3.41)	2.57 (0.72)
Jaguarundi	*Puma yagouaroundi*	5	13	0.32	0.22	0.39	0.91 (0.10)	0.80 (0.21)	1 (#)	—
Ocelot	Leopardus pardalis*	16	12	0.50	0.70	0.36	0.72 (0.01)	0.72 (0.11)	0.71 (0.18)	−1.73 (0.85)
Puma	*Puma concolor*	2	1	0.05	0.09	0.03	—	—	—	—
Greater grison	*Galictis vittata*	1	2	0.05	0.04	0.06	—	—	—	—
Tayra	*Eira barbara*	2	0	0.04	0.09	0.00	—	—	—	—
Crab-eating raccoon	Procyon cancrivorus*	4	7	0.20	0.17	0.21	0.42 (0.20)	0.37 (0.22)	0.46 (0.23)	0.36 (0.95)
Coati	*Nasua nasua*	2	0	0.04	0.09	0.00	—	—	—	—
White-tailed deer	Odocoileus virginianus*	6	12	0.32	0.26	0.36	0.57 (0.17)	0.45 (0.19)	0.65 (0.21)	0.79 (0.95)
Red-brocket deer	*Mazama spp*	0	1	0.02	0.00	0.03	—	—	—	—
Collared peccary	*Pecari tajacu*	1	1	0.04	0.04	0.03	—	—	—	—
Spiny rat	*Proechimis spp*	21	2	0.41	0.91	0.06	0.40 (0.06)	0.88 (0.07)	0.06 (0.04)	−4.21 (1.21)
Agouti	*Dasyprocta fuliginosa*	18	0	0.32	0.78	0.00	0.69 (0)	0.67 (5.78)	0	−4.75 (1.13)
Lowland paca	*Cuniculus paca*	23	1	0.43	1.00	0.03	0.43 (0.07)	1 (#)	0.03 (0.03)	—
Capybara	*Hydrochaerus hydrochaeris*	8	3	0.20	0.35	0.09	0.20 (0.05)	0.36 (0.10)	0.09 (0.05)	−1.62 (0.77)
Coendu	*Coendu spp*	1	0	0.02	0.04	0.00	—	—	—	—
Squirrel	*Sciurus spp*	9	0	0.16	0.39	0.00	—	—	—	—
Common opossum	*Didelphis marsupialis*	22	12	0.61	0.96	0.36	0.49 (0.27)	0.80 (3.32)	0.27 (1.69)	−3.62 (1.10)
Four-eyed opossum	*Philander opossum*	1	0	0.02	0.04	0.00	—	—	—	—

*Overall occupancy model did not converge, therefor $\hat{\Psi }$ for study in these species is from null model [*Ψ*(.), *p*(.)], and for each habitat from *Ψ*(cov), p(.). Therefore, parentheses is SE and not SD.

— = Data Deficient. Models using species with less than 4 detections resulted in convergence issues and high uncertainty.

# = SD extremely high.

In oil palm plantations, only two species occupied more than 70% of the sites with the giant anteater being the most widely distributed species (naïve occupancy 91%) followed by the fox (naïve occupancy = 79%; Table 1). Jaguarundi (*Puma yagouarondi*) was detected at nearly 40% of the oil palm sites, but the remaining species detected in the oil palm plantations were rare. Nine out of 17 (53%) species detected within oil palm sites occupied less than 10% of the sites (Table 1). In forested sites, however, seven species occupied more than 70% of the sites: giant anteater, lesser anteater, nine-banded armadillo–here after armadillo (*Dasypus novemcinctus*), ocelot, spiny rat (*Proechimis spp*), agouti (*Dasyprocta fuliginosa*), lowland paca–here after paca (*Cuniculus paca*), and common opossum. Within forested sites, seven out of 23 species (30%) occupied less than 10% of the sites.

### Effects of habitat type on species habitat use

Only 12 of the 23 identified species had sufficient detection records for analyses. Based on their weight of evidence (Σ*ω*_***i***_), an effect of habitat type on habitat use (*Ψ*) was detected for only four of these species: naked-tailed armadillo (*Cabassous unicinctus*), capybara (*Hydrochoerus hydrochaeris*), common opossum, and spiny rat; all displaying a strong negative effect of oil palm (β coefficient) on the probability of habitat use (Table 1). We observed no effect of habitat type (i.e. support for the model based on constant occupancy was higher) in the six remaining species: ocelot (Σ*ω*_***i***_ = 1), fox (Σ*ω*_***i***_ = 1), crab-eating raccoon (*Procyon cancrivorus*)–here after raccoon (Σ*ω*_***i***_ = 0.70), white-tailed deer (*Odocoileus cariacou*; Σ*ω*_***i***_ = 0.71), and to a lower extent nine-banded armadillo (Σ*ω*_***i***_ = 0.61), and jaguarundi (Σ*ω*_***i***_ = 0.55). However, the models suggested that habitat type had an important influence on the detection probabilities of many species (Fig. S1, Table S2).

There was no single best model explaining the effect of habitat type for either species of anteaters. The weight of evidence (Σ*ω*_***i***_) for the model that had constant habitat use [*Ψ* (.), *p*(hab)] received similar support as the model containing habitat type [*Ψ* (hab), p(hab)] (Table S1). However, the two anteaters species showed different response directions for the effect of habitat type, with occupancy for giant anteater displaying a positive association with oil palm (β = 2.12 ± 2.60 SE), whereas occupancy for the lesser anteater exhibited a negative association with oil palm (β = −1.64 ± 0.97SE) (Table 1).

### Habitat use probability between oil palm and riparian forest

**Figure 2:**
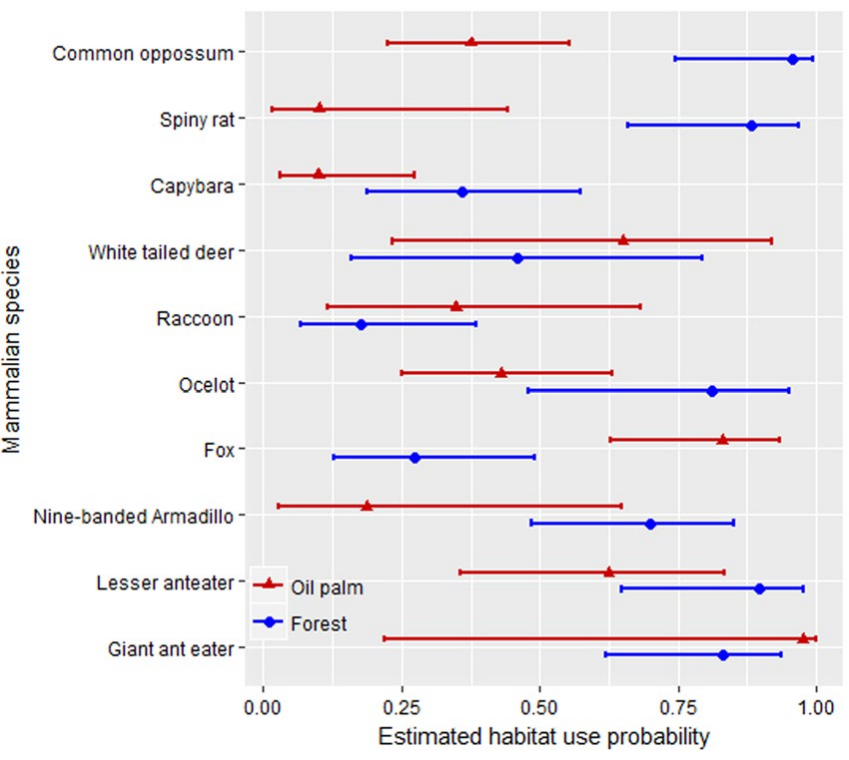
Estimated habitat use probability ($\hat{{\boldsymbol{\Psi }}}$) of selected species between oil palm and riparian forest in the Llanos region of Colombia. Model ***Ψ*** (cover), p(cover) was used to compare across species. Note: only 10 of the 17 shared species were suitable for analysis (>3 detections per habitat). For rare species or those with convergence issues see Table 1. Some common names have been shortened to facilitate interpretation, see Table 1 for scientific and common names. Confidence intervals are shown to assess the precision of the estimated occupancy, caution is advised if interpreted as “significance” testing (i.e. overlap of confidence intervals do not necessarily suggest no evidence of difference (“not significant”; see Helman and Stern 2006, and http://mikemeredith.net/blog/1303_Comparison_of_confidence_intervals.htmo).

Seventeen mammal species occurred in both oil palm and forest. However, only 10 species had sufficient sample sizes (i.e. >3 occurrences) to compare estimated habitat use probabilities between both ecosystems – Ψ (habitat), *p*(habitat). From these species, six had lower probability of oil palm use when compared to forest: capybara, spiny rat, armadillo, common opossum, ocelot and lesser anteater. Moreover, the first three of those species were the least likely species to use oil palm, according to the estimated probabilities of habitat use $(\hat{{\Psi }})$ (Fig. 2). Further, for these six species, habitat use was on average 2.2 (range 1.4–3.6) times higher for forest than oil palm. In contrast, fox, raccoon, white-tailed deer and giant anteater were the most likely species to use or occupy oil palm plantations, with habitat use probabilities being on average ~2 times greater there than inside forest (range 1.4–3.0) (Fig. 2).

Although the estimated habitat use of jaguarundi was four times greater in oil palm than in forest, these estimates were associated with high SEs (inside oil palm). For the eight species that were so infrequently detected that the models did not converge, naïve occupancy suggested larger differences between oil palm and forest. For example, the paca was only detected at one site inside oil palm but occupied all forested sites. Similarly, spiny rats occupied almost all forested sites and were detected in only two oil palm sites. However, other species were equally rare in both habitats (e.g. puma [*Puma concolor*], grison [*Galictis vittata*]). Estimated detection probabilities were below 0.2 for 11/13 species, particularly inside oil palm (7/11 species), while white-tailed deer, raccoon, fox and jaguarundi exhibited higher detection probabilities inside oil palm (Fig. S2).

### Effects of variables on habitat use within oil palm sites

**Figure 3:**
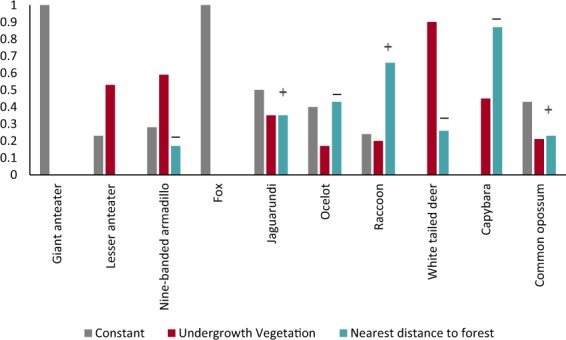
Cumulative Akaike weight, (Σ*ω*_i_), for support of the influence of undergrowth vegetation and distance to nearest forest patch on the occupancy of selected medium and large mammals inside oil palm plantations in Colombia. Positive or negative signs indicate the direction of the effect for distance to nearest patch. Undergrowth vegetation had a positive effect in all species. *Σω_i_ calculated for top-ranked models Δ ACIc < 2.

**Figure 4:**
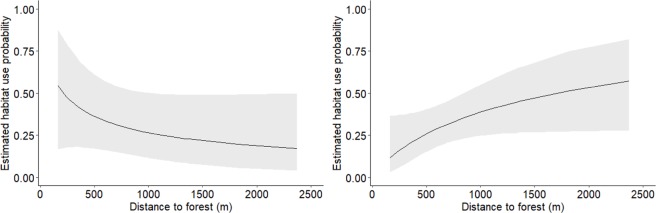
Estimated effect of distance to nearest forest on habitat use probability of selected species. Figure represents the effect on different species that showed strong evidence for the influence of distance to forest with left panel representing the cumulative effect on armadillo, ocelot, white-tailed deer, and capybara, whereas right panel represents the positive cumulative effects on jaguarundi, raccoon, and common opossum. Note: species were grouped to make predictions more precise. Individual species develop wide confidence intervals due to sparse data inside oil palm, which limits interpretation of the effects of the variable. Selection of species was based on Σω_i_ from Fig. 3.

Ten species had sufficient frequency of detections to examine occupancy within oil palm. We found strong evidence in support of no effect of understory vegetation or distance to nearest forest on habitat use in giant anteater and fox (Table 2, Fig. 3). Undergrowth vegetation had a positive effect on the habitat use of the remaining eight species with particularly strong support (Σ*ω*_i_ > 0.5) for the lesser anteater, armadillo, and white-tailed deer. Distance to nearest forest patch had the greatest influence on raccoon and capybara habitat use (Σ*ω*_i_ > 0.66), with positive and negative associations with increasing distance, respectively. Ocelot too was strongly negatively affected by increasing distance to forest. However, modeling for habitat use of the ocelot also revealed a similar level of support for models including constant habitat use (Fig. 3). Four species showed a negative relationship between distance to forest and habitat use probability (armadillo, ocelot, white-tailed deer and capybara), whereas three species showed a positive relationship (jaguarundi, raccoon, common opossum) (Figs 3 and 4). For jaguarundi and the common opossum both distance to forest and presence of undergrowth vegetation had the same level of support and direction of the effect, but their null models had higher support, suggesting limited influence of these variables on their habitat use (Fig. 3).

**Table 2:** Model selection results evaluating the effect of understory vegetation (veg) and distance to forest patch (dist) on estimated habitat use (*Ψ*) and detection probabilities (p) for selected species within oil palm plantations in the eastern plains of Colombia (n = 33 sites). Only the top supported models (Δ AICc < 2) are shown.

Species/Model	Δ AIC	AIC *w*	k	−2 log like	Intercept	SE	Beta_1_	SE	Beta_2_	SE
**Giant anteater**										
Ψ(.), *p*(.)	0.00	1.00	2	298.29						
**Lesser anteater**										
Ψ(veg), *p*(.)	0.00	0.53	3	165.42	−0.73	0.76	2.03	1.14		
Ψ(.), *p*(.)	1.62	0.23	2	169.47						
**Nine-banded armadillo**										
Ψ(veg), *p*(.)	0.00	0.42	3	36.02	−23.65	4.38	22.74	4.38		
Ψ(.), *p*(.)	0.79	0.28	2	38.81						
Ψ(veg + dist), *p*(.)	1.78	0.17	4	35.80	−26.10	4.77	28.33	4.57	−0.50	0.79
**Fox**										
Ψ(.), *p*(veg)	0.00	1.00	3	274.77						
**Jaguarundi**										
Ψ(.), *p*(.)	0.00	0.50	2	118.25						
Ψ(veg + dist), *p*(.)	0.71	0.35	4	113.93	−12.47	9.14	22.13	#	1.79	1.37
**Ocelot**										
Ψ(dist), *p*(.)	0.00	0.43	3	104.74	46.00	#	−6.44	#		
Ψ(.), p(.)	0.16	0.40	2	107.33						
Ψ(veg), *p*(.)	1.87	0.17	3	106.61	0.61	1.57	25.57	#		
**Crab-eating Raccoon**										
Ψ(dist), *p*(.)	0.00	0.45	3	76.94	−11.34	5.21	1.65	0.83		
Ψ(.), *p*(.)	1.23	0.24	2	80.17						
Ψ(veg + dist), *p*(.)	1.59	0.20	4	76.53	−12.72	5.41	0.83	1.29	1.77	0.82
**White tailed deer**										
Ψ(veg), *p*(.)	0.00	0.64	3	119.14	−1.88	1.13	3.28	1.73		
Ψ(veg + dist), *p*(.)	1.79	0.26	4	118.33	6.56	17.90	3.51	2.81	−1.31	2.76
**Capybara**										
Ψ(veg + dist), *p*(.)	0.00	0.45	4	39.58	−4.57	16.54	22.07	14.28	−3.20	1.68
Ψ(dist), *p*(.)	0.10	0.42	3	41.68	16.44	10.01	−3.10	1.74		
**Common opossum**										
Ψ(.), *p*(.)	0.00	0.43	2	162.84						
Ψ(dist), *p*(.)	1.24	0.23	3	161.65	−4.58	3.46	0.63	0.53		
Ψ(veg), *p*(.)	1.38	0.21	3	161.79	−1.05	0.68	0.81	0.81		

Notes: Δ AICc: difference in AIC values between each model with the lowest AIC model (best model); AICω*:* Akaike weight.; k: number of parameters in the model; SE: standard error. Understory vegetation is a binary covariate with 0 = clean or low understory vegetation (the intercept), and 1 = medium to high understory (beta), nearest distance to forest in log10, # = high standard errors, this does not affect the direction or effect of the untransformed beta estimate (Hines, 2006).

## Discussion

We found that the mammal community in the oil palm producing region of the Llanos in Colombia is composed mostly of generalist species, with restricted distributions and very low detection probabilities (especially for most species within oil palm). These results are supported by the findings of other research on different taxonomic groups in Colombia that have reported a negative relationship between oil palm and biodiversity metrics such as richness, occupancy, and abundance^32,34,37,42^. However, eight out of twelve species were not significantly influenced by habitat type, which suggests that some species are able to use and move across the oil palm matrix.

Understanding the probability that species use human altered environments or agroecosystems helps to evaluate the extent of impacts and conservation prospects for wildlife-friendly agriculture. We found that oil palm is more likely used by generalist mesopredators, anteaters, and deer, whereas seed dispersers (e.g. paca and other rodents), some omnivores (e.g. armadillos) and marsupials were less likely to use oil palm, suggesting they are more sensitive to forest conversion to oil palm. As such, our findings highlight the importance of retaining secondary forest in agroindustrial landscapes to support terrestrial mammal occurrence. These findings reinforce the critical importance of riparian forests to maintain biodiversity, and support previous findings in different geographic locations and diverse taxa^32,43,44^. In the following paragraphs, we briefly discuss the possible mechanisms behind the most conspicuous patterns detected in the western Llanos, where contrary to other regions, ~80% of oil palm expansion has taken place on reclaimed pasture and crop lands^20^.

### Anteater and armadillo species habitat use

The giant anteater commonly used oil palm. This pattern clearly contrasts with the results of Mendes-Oliveira *et al*.^45^, who found negative associations for this species in the Brazilian Amazon oil palm plantations. While the giant anteater is categorized as Vulnerable by the International Union for Conservation of Nature–IUCN^46^, we found they are widely distributed throughout both oil palm plantations and forest habitats. This habitat flexibility corresponds with other research suggesting that intermediate human disturbance and resultant habitat heterogeneity seem to be favorable to giant anteaters^47^. This pattern suggests that food availability for giant anteaters is not a limiting factor inside oil palm plantations, a contention supported by the relatively high diversities and abundances of ant species found in this crop^32,48^. On the other hand, jaguars (*Panthera onca*) have a strong preference for giant anteaters in their diet (i.e. 75% of the biomass consumed) in central Brazil^49^ and capture frequencies of giant anteaters tend to increase when jaguars are absent^47^. Therefore, it is also possible that the apparent absence (no detections) of jaguars in the current study area could, in part, facilitate the high occurrence of giant anteaters in the area. The fact that giant anteaters tend to be more common in savanna-like ecosystem than rainforest^50^ may also contribute in generating this difference between the Amazon and the Llanos oil palm landscapes.

Contrary to the giant anteater, the lesser anteater displayed a preference for forested habitats. This is despite the fact that the giant and lesser anteaters display similar dietary requirements (i.e. obligate insectivores)^50^. Like the giant anteater, lesser anteaters forage terrestrially, however they also use trees for nesting and foraging^51,52^, which may in part explain their preference for forest. Further, lesser anteaters feed mainly on arboreal ants and termites, while giant anteaters mainly eat terrestrial ant species and tend to eat less termites^53^. Oil palm trees typically lack termite nests (LEP pers. obs.), so it is probable that the availability of food resources for the lesser anteater is reduced inside oil palm plantations which would negatively impact their occurrence within them. Although the lesser anteater was able to cross oil palm plantations, their occurrence was reduced as the distance to forest increased, suggesting a scale-dependent effect of oil palm permeability for the species.

As with the lesser anteater, oil palm does not appear to offer enough resources for species such as armadillos. This was reflected by the low probabilities of detection in oil palm sites. Naked-tailed armadillos, for example, prefer complex vegetation associated with riparian forests running through savanna type habitats^54^. Similarly, although the nine-banded armadillo is commonly detected in sites associated with various land-use matrices (e.g. agricultural landscapes) in the Neotropics^8,55^, reduced leaf litter and decomposing soil inside oil palm plantations most likely limits food resource availability for this species (e.g. soft-bodied invertebrates, fruits, seeds, and small vertebrates^56,57^. High rates of disturbance associated with farming activity (e.g. harvesting, motor vehicles etc.) and vulnerability to mesopredators (e.g. fox, ocelots) could also play an important role in limiting use of oil palm habitat by nine-banded armadillos.

### Herbivore, frugivore, and granivore mammal species habitat use

Paca and agouti are two medium-sized frugivores and important seed dispersers in the study area. These species were detected exclusively within forested sites and commonly encountered within them, most likely because oil palm does not provide the necessary fruit/seed resources for them. Additionally, their limited dispersal ability and small home ranges^58,59^ reduce their probability to cross the oil palm matrices in search of riparian forest patches. Considering the important role these species play in forest maintenance and regeneration^60,61^, their vulnerability to conversion of forested ecosystems to oil palm plantations could alter ecosystem processes (also see^37^). Similar patterns were shown for other seed dispersers such as spiny rats, which exhibited high habitat use of forested sites compared to oil palm plantations, and squirrels that were also only ever detected in forest sites. As such, if oil palm expansion replaces native vegetation in the Llanos, it may threaten seed dispersing mammalian fauna, and as a consequence threaten the regenerative capacity of the local forested ecosystems.

### Top and mesopredator mammal species habitat use

One top predator and eight mesopredators (seven carnivores and common opossum) were detected in the study. Individual species within this group exhibited varying responses to the oil palm landscape. Pumas (*Puma concolor*) were rarely detected (see^62^), which limit further inferences, but this finding corresponds with other research that suggest that top predators are able to traverse oil palm plantations (e.g. pumas, jaguar and tiger)^45,63–65^. These findings, however, do not constitute evidence that oil palm is suitable as permanent territories for apex predators. Moreover, in all of the above cases, oil palm sites were relatively close to forest habitats, which suggests that the retention of natural forest is critical to maintain top predator occurrence on the landscape (see also^66,67^).

The higher habitat use probabilities within oil palm plantations for some mesopredators (e.g. fox, jaguarundi, raccoon) confirms their ecological flexibility and thus ability to occupy several types of land-cover/use including agroecosystems^10,50,68^. Fox, in particular, clearly preferred oil palm habitats which is consistent with previous findings^37,42,45^. Mechanisms underlying this ecological flexibility may include their omnivorous diet and opportunistic feeding behaviors that allows them to consume a wide range of fruits, insects, amphibians, reptiles, crustaceans and rodents^50,69,70^. Further studies are required to elucidate whether fox habitat use is also influenced by oil palm fruit, as previous research suggests foxes may consume this fruit as they have been recorded consuming similar palm fruits and seeds^69,71^.

Although there is a lack of information on small rodents within oil palm plantations in Colombia, they have been reported as a pest in Southeast Asian plantations^72^. Since the diet of mesopredators often includes a high proportion of small rodents^68,69^, it is likely that the potential availability of rodents could increase oil palm use by this group of carnivores. Apart from rodents, the presence of other potential prey items (such as armadillos, reptiles and invertebrates) reported inside oil palm^42,50,68^, may also play a crucial role in high oil palm use by mesopredators.

Similar to the jaguarundi, ocelots are obligate carnivores highly tolerant of anthropogenic systems^8,45,73,74^. However, ocelot displayed higher use of forest sites than of oil palm, while jaguarundi preferred oil palm. This difference in habitat selection between the species could be related to spatial segregation, which has been previously suggested as a way to minimize competition among mesocarnivores^75^. It is unclear, though, whether ocelots may use oil palm for passage rather than as permanent territories for hunting. Dietary studies would help to elucidate whether the coexistence of these two species is achieved through resource partitioning.

### Habitat use within oil palm plantations

Within oil palm plantations the existence of understory vegetation was related to increased habitat use by the majority of species, and tended to have a stronger effect than the distance a site was from forest. This finding may occur because understory vegetation can increase habitat heterogeneity inside plantations thereby promoting diversity and trophic interactions at different scales (reviewed by^76^). Undergrowth vegetation in the oil palm plantations in this study was found to be especially important for deer, which suggests these resources could be important in terms of foraging or sheltering from predators or hunters^77^.

The low contribution of distance to forest in determining the habitat use of some species within oil palm plantations could reflect their adaptability to oil palm. This hypothesis corresponds with those of previous studies showing attributes such as quality (in terms of resource availability) and cover type seemed to be more important for species occurrence than measures of isolation from forest patches^7,8^. Our finding, however, contrasts with that of Yue *et al*.^31^ who found that distance to forest was the most important factor in determining occupancy of the mammalian assemblage in Sabah (Malaysian Borneo). It is difficult to make direct comparison between this study and Yue *et al*.^31^ since they compared distance to forest patch with canopy cover, oil palm height and their interaction, but not vegetation inside plantations as we did.

An important exception in the effect of distance to forest was the capybara whose habitat use was 3.6 times greater in riparian forest than in oil palm, and decreased with increasing distance to nearest forest patch, with detections never occurring further than 357 m from remnant forest. This finding supports the notion that capybaras are dependent on forested habitats and waterways, despite also being a common species in open natural savannas elsewhere in the country^78,79^.

The relationship between habitat use and proximity to forest varied among species. Four out of seven species showed a negative relationship between distance to nearest forest patch and habitat use (armadillo, ocelot, white tailed deer, and capybara). This relationship suggests that these species may freely move inside oil palm but never occur far from the native forest. Similarly, Menses-Oliveira *et al*.^45^ identified that the median distance from the nearest forest edge for any mammal detected in their oil palm study was 960 m. In our study, the average distance from the nearest forest edge for rare species (those with insufficient data to model; <3 sites; e.g. greater grison, puma, red brocket, peccary, paca) was 430 m (SD 169 m), supporting the contention that rare species that occur in oil palm are restricted to locations close to natural forest.

Further, species with a negative association with distance to the forest showed very low probabilities of habitat use beyond 1000 m distance from forest edges. In other words, there are likely spillover effects from forests to oil palm plantations, though these are limited to relatively short distances from the forest. It is important to note that the maximum distance to the nearest forest in the study area was ~2400 m. Therefore, it is unknown whether these effects could be more accentuated when the closest forest patches are at greater distances. In this sense, Pardo *et al*.^80^ found potential tipping points or thresholds on species composition of some species as oil palm cover percentage extended for more than half of the landscapes (scales of 2 km^2^ approximately). This clearly suggests that larger areas of oil palm isolated from nearby forest may decrease the probability of mammals occupying oil palm and therefore may reduce the ability to move across the landscape.

Clearly, the majority of the mammals in the study area are not likely to use oil palm as a permanent habitat compared to forested ecosystems (see also^37^). However, to assess the direct impacts of oil palm cultivation on Colombian biodiversity, it is important to consider the effects of previous land-use types, as well as hunting history on the current patterns of diversity (see^81,82^). Oil palm in Colombia has been implemented mostly in lands previously modified for other human uses–particularly in the last two decades^16,20^. Therefore, the responses shown in this study could be influenced by the accumulation of historical processes of land-use changes (e.g. rice, cattle pastures) and not solely the effect of oil palm. It is likely that the species now occupying oil palm plantations are “survivors” (*sensu* Prugh *et al*.^7^) that are more readily adaptable to this agricultural ecosystem or that are attracted to this “new” ecosystem. The inability to detect more sensitive species (e.g., tapirs [*Tapirus terrestris*], jaguars, giant armadillo [*Priodontes maximus*]) in the study area may support this hypothesis.

The production of oil palm in already transformed lands may positively or negatively affect some of the relationships between resilient species across this new anthropogenically transformed landscape. For instance, raccoons and foxes tend to prefer forest rather than pastures^83^, while in the study area they tended to prefer oil palm over forest. Therefore, future research is required to monitor the dynamics taking place among these assemblages and compare the effects of other crops on mammalian communities with that of oil palm (e.g. pasture, rice, soy etc.). Future studies will also benefit from evaluating the influence of proximate land uses and configuration of the entire landscape mosaic, as well as using multiple approaches for assessing functional connectivity within oil palm landscapes (e.g. using Graph or Circuit theory).

## Methods

### Study area

This study was conducted in the Llanos region of Colombia. The ~2,000 km^2^ study area is located in the department of Meta, in rural land surrounding the towns of Restrepo, Cumaral, Cabuyaro, Acacias, Castilla la Nueva and San Carlos de Guaroa. Elevations in the area range between 194–394 m (Fig. 1). This area has a long history of human activity and contains a heterogeneous pattern of land-cover including natural ecosystems of differing successional status interspersed by human land uses, such as livestock grazing and crop production. The predominant land-use is oil palm followed by cattle grazing and then other minor crop systems (see^27^). The remnant native vegetation in the region consists of secondary riparian forest strips (known locally as gallery forest) of differing sizes and ages, some of which experience seasonal flooding.

### Survey design

We sampled 33 sites inside oil palm plantations (hereafter: oil palm) and 23 sites within riparian forests (hereafter: forest) with sampling effort proportionate to the spatial extent of these habitat types within the study area (Fig. 1). Sites within each habitat were a minimum of 2 km apart to ensure the spatial independence of samples. The criterion that sites be separated by >2 km was imposed as it exceeds the minimum recommended inter-site distance for inventories of terrestrial mammals in the Neotropics^84–86^ and encompasses the average expected diameter of the home ranges of the most common species found in the study area^26,42^. Surveys in oil palm plantations were restricted to those planted before 2006 to avoid the confounding influence of plantation age. All surveys were conducted during the dry season, between September 2014 and January 2016.

### Camera trapping

We deployed seven Reconyx HC500 HyperfireTM digital camera traps at each site (sampling unit) to detect terrestrial mammals (>0.5 kg). This sampling design of seven camera traps was found in a pilot study conducted in the study area (Pardo *et al*. not publ.) to improve estimates of the sampling completeness when compared with the traditional practice of using a single camera per site^87^. In the forest, camera traps were spaced ~250 m apart along transects and were set close to animal trails and natural funnels where possible. Due to the homogeneous nature of oil palm plantings, cameras in this land-use type were placed at similar spacing as in the forests but arranged in a zigzag pattern in an attempt to maximize spatial coverage. We pooled the data derived from the seven cameras at each site into a single sample and used this as the sample unit for estimating species occupancy (see below).

All cameras were fixed to trees or wooden poles with a steel cable (Python^TM^, US) and were configured to the following criteria: high-sensitivity sensor, a 1-second interval between consecutive pictures (3 per trigger), no delay or quiet period between triggers. Cameras were installed a minimum distance from the potential path of the animal (e.g. trails) of 1 m and 25–30 cm height, depending on the terrain. Cameras were active for 30 to 40 days without baiting. Only terrestrial mammals were considered for analyses and were identified using the most recent taxonomic classification of Colombian mammals^22^.

### Habitat use and detection probability

To evaluate the effects of oil palm on habitat use and detection probabilities of terrestrial mammalian species, we used a single season likelihood-based occupancy modeling protocol as developed by MacKenzie *et al*.^41^. This framework allows for the determination of factors that affect the distribution or habitat use of a particular species based on detection/non-detection data along temporal replicates (i.e. detection histories) at different sites. Furthermore, occupancy analyses explicitly account for imperfect detection (*p* < 1) and therefore facilitate evaluations of changes in detection probabilities and likelihood of species’ use of particular habitats (e.g.^41,88^). Since we were interested in the entire terrestrial mammal community, the criteria and assumptions about independence between sites within the occupancy framework were flexible (i.e. sites might not be closed to changes in occupancy). Thus, occupancy (*Ψ*) in this study is interpreted and referred to as habitat, or site, use probability^41^. Detection histories were constructed using the compiled photographs from eight survey occasions, each formed by the combination of five camera survey days across all seven cameras per site.

We aimed to answer two questions. First, we evaluated whether habitat type (oil palm versus forest) was a determinant driver for habitat use (*Ψ*) and detection probability (*p*) across the study area (n = 56 sites). Second, we selected only those sites within oil palm (n = 33) to assess how two habitat characteristics of this monoculture influenced species habitat use: distance to nearest forest patches (m) (log_10_ transformed) and understory vegetation (high = 1 or low = 0). Due to relatively small sample size and limited variation within oil palm plantations, we could not include other features of the structure of the plantations such as height or canopy cover. However, since the oil palm structure is evidently homogeneous, these factors do not likely influence medium to large-sized mammals habitat use, as has been shown for other biodiversity measures (e.g.^37^).

To assess the influence of the explanatory variables in the above questions, we used a model selection framework based on Akaike’s Information Criterion corrected for small sample size (AICc)^89^. We evaluated the models for goodness-of-fit against 1,000 simulated bootstrap datasets^90^. All models within Δ AICc < 2 were considered to have substantial support as the most likely factors influencing *Ψ* and/or *p*^89^. We used the software program PRESENCE 3.1^91^ for all analyses of occupancy and model selection. Models that did not converge were not included in the model selection process. In cases where overdispersion was detected (which occurred only for the crab-eating fox), QAICc was used for model selection. The relative importance of the effect of each variable on species occupancy was assessed by summing the cumulative Akaike weight of evidence (Σ*ω*_*i*_) of those models containing the variable, using a cutoff of Δ AICc < 2. We also used a model-averaging approach to estimate an overall value of occupancy for the entire study area and for each habitat type.

To evaluate the effect of habitat type, we constructed four models allowing habitat use (i.e. occupancy - *Ψ*) and detection probabilities (*p*) to vary according to habitat type. If the null model for *Ψ* (i.e. constant habitat use) appeared among the top candidate models with sufficient Σ*ω*_***i***_, then we concluded that habitat had no strong (“significant”) effect on the occupancy, and hence oil palm plantations had no apparent influence on determining habitat use probabilities. Further, to clearly understand how habitat use of each species differed between oil palm and forest, we used the model *Ψ* (habitat), *p*(habitat) as a unifying criterion. This model provides information on the probabilities of species using any of both habitats while accounting for likely differences in detection probabilities due to the differences in habitat structure. This model was also commonly among top-ranking models in six of the 12 species (Table S1). Preliminary analyses showed that models constructed for species detected in less than three sites resulted in model convergence issues. Estimation of *Ψ* and *p* was not possible for these species and naïve occupancy is reported instead (i.e. the proportion of sites where species were detected without accounting for imperfect detection).

To understand the effect of the selected variables on habitat use of species within oil palm, we first evaluated the likely effects of undergrowth vegetation on detection probabilities (*p*) (i.e. the animal can be present but not detected due to vegetation surrounding the camera). To do this, we allowed *p* to be constant or vary according to undergrowth vegetation while maintaining the habitat use parameter–*Ψ* identified in the global model (all variables) [i.e. *Ψ* (undergrowth + distance to forest), *p*(constant)] vs *Ψ* (undergrowth + distance to forest), *p*(undergrowth)]. The best model for *p* was then used in the subsequent model selection of *Ψ*. Untransformed beta coefficients from these models were used to evaluate the direction and magnitude of the effect of the variables on *Ψ* estimates, which are presented for each of the top-ranking models. We combined the detection history of species with similar direction in response to distance to forest (only those with strong evidence, Δ AICc < 2) to improve the precision of the estimates inside oil palm due to the low occurrences of species and to facilitate the visual interpretation of the results.

## Conclusions

We aimed to compare the habitat use and detection probabilities of terrestrial mammals between oil palm plantations and riparian forests in the Colombian Llanos. We found a reduction in diversity of terrestrial mammals associated with low probabilities of habitat use of oil palm plantations by most species. However, some species such as giant anteater, omnivorous mesopredators, and white-tailed deer seemed to prefer oil palm over riparian forest. Due to the variable, species-specific responses we report, caution must be exercised against a “one size fits all” solution and generalizations across all mammal species. Our results suggest that oil palm plantations in Colombia could be made more suitable for the majority of the resident mammal species with the provision of undergrowth vegetation and by ensuring remnant forests are retained in their proximity. The high habitat use found in forested habitats, even in those species positively associated with oil palm cover (e.g. giant anteater) implies that oil palm plantations alone would be insufficient for the persistence of wild mammals. Therefore, a heterogeneous landscape with interspersed native vegetation is required to maintain connectivity and habitat use by mammals across the oil palm matrix in Colombian landscapes. To fully understand the impacts of oil palm in Colombia it is important to recognize the regional landscape context and the history of land-uses prior to the implementation of oil palm.

## Supplementary information

Supplementary information

## Data Availability

The datasets generated during and/or analysed during the current study are available in the espaces repository, https://espaces.edu.au/lepardov/scientific-report-publication_repository.
