# Supplementary material for: Land management strategies can increase oil palm plantation use by some terrestrial mammals in Colombia
Source: Sci Rep. 2019 May 24;9:7812. doi: 10.1038/s41598-019-44288-y (PMC6534675; doi:10.1038/s41598-019-44288-y)
Supplement: Supplementary file 1 — Supplementary information [file 41598_2019_44288_MOESM1_ESM.pdf]

Supplementary information

Land management strategies can increase oil palm plantation use by some terrestrial mammals in Colombia

Lain E. Pardo[1,2]*, Mason J. Campbell[2], Michael V. Cove[3], Will Edwards[2], Gopalasamy Reuben Clements[4,5] and William F. Laurance[2]

*lepardov@gmail.com

[1] School of Natural Resource Management, Nelson Mandela University, George Campus, Madiba Drive 6560, South Africa

[2] Centre for Tropical Environmental and Sustainability Science (TESS), College of Science and Engineering, James Cook University, Cairns, Queensland, 4878 Australia.

[3] Department of Applied Ecology, North Carolina State University, Raleigh, North Carolina, 27695, USA

[4] Department of Biological Sciences, Sunway University, 47500 Bandar Sunway, Selangor, Malaysia

[5] Rimba, Jalan Kiara 5, 50480 Kuala Lumpur, Malaysia

Fig S1. Detection probability($\hat{p}$)for most common species across oil palm dominated landscapes in Colombian Llanos. Error bars indicate confidence intervals.

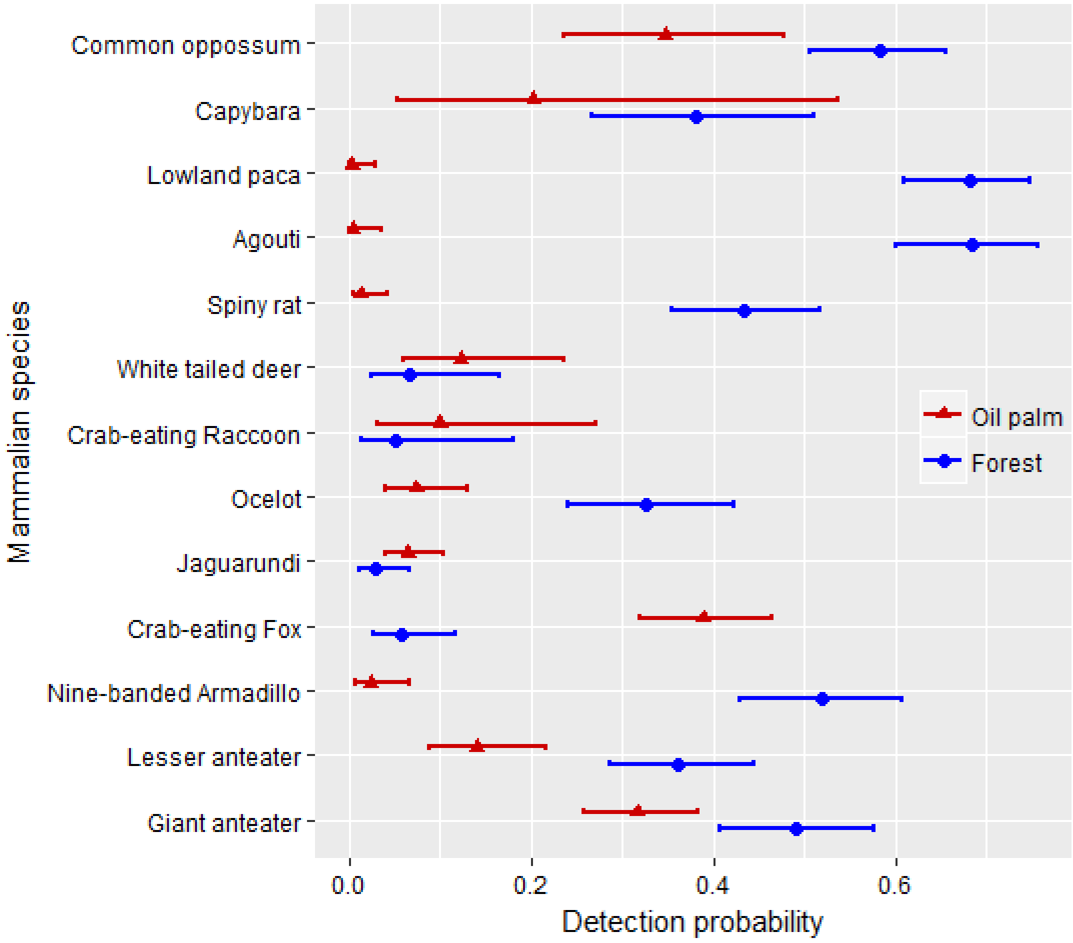

Table S1. Model selection (Δ AICc <2) evaluating the effect of habitat type (hab) on habitat use probabilities (Ψ) and detection probabilities (p) for selected mammal species*. Habitat type is a binary variable where 0=forest (intercept) and 1= oil palm plantation (β), a negative value of the corresponding β coefficient suggest preference for forest and vice versa.

**Model**	**AIC**	**Δ AICc**	**AIC *w***	***k***	**β (SE) Oil palm**
**Giant anteater**					
Ψ(.),p(hab)	526.26	0	0.501	3	
Ψ(hab),p(hab)	526.32	0.06	0.4862	4	2.12 (2.60)
**Lesser anteater**					
Ψ(hab),p(hab)	391.21	0	0.4979	4	-1.64 (0.97)
Ψ(.),p(hab)	391.32	0.11	0.4713	3	
**Nine-banded armadillo**					
Ψ(.),p(hab)	242.34	0	0.6103	3	
Ψ(hab),p(hab)	243.25	0.91	0.3872	4	-2.31 (1.15)
**Naked-tailed armadillo**					
Ψ(hab),p(.)	110.55	0	0.7612	3	-26.48 (#)
**Crab-eating fox**					
Ψ(.),p(hab)	365.87	0	0.9965	3	
**Jaguarundi**					
Ψ(.),p(.)	170.78	0	0.5548	2	
Ψ(hab),p(.)	171.22	0.44	0.4452	3	27.03 (#)
**Ocelot**					
Ψ(.),p(.hab)	294.61	0	0.9998	3	
**Crab-eating raccoon**					
Ψ(.),p(.)	124.6	0	0.4516	2	
Ψ(.),p(hab)	125.76	1.16	0.2529	3	
**White-tailed deer**					
Ψ(.),p(.)	190.76	0	0.3984	2	
Ψ(.),p(hab)	191.27	0.51	0.3087	3	
Ψ(hab),p(.)	192.25	1.49	0.1891	3	0.79 (0.95)
**Capybara**					
Ψ(hab),p(.)	166.95	0	0.5784	3	-1.69 (0.95)
Ψ(hab),p(hab)	168.66	1.71	0.246	4	-1.61 (0.96)
**Spiny rat**					
Ψ(hab),p(hab)	260.92	0	0.5125	4	-4.20 (1.22)
Ψ(.),p(hab)	261.59	0.67	0.3666	3	
**Common opossum**					
Ψ(hab),p(hab)	404.11	0	0.969	4	-3.62 (1.10)

Notes: Δ AICc = difference in AIC values between each model with the lowest AIC model (best model); AICω = Akaike weight.; k = number of parameters in the model; SE: standard error. Habitat type, a binary covariate with 0 =

forest (intercept) and 1= oil palm (beta). # = high standard error, so species-specific occupancy estimates are imprecise. However, direction of the effect is not affected (Hines et al 2006). Only species with sufficient data to conduct modeling are shown (i.e. at least 4 detections per habitat). Refer to naïve occupancy in Table 1 for rare species and scientific names.
